# Long intergenic non-coding RNAs regulate human lung fibroblast function: Implications for idiopathic pulmonary fibrosis

**DOI:** 10.1038/s41598-019-42292-w

**Published:** 2019-04-15

**Authors:** Marina R. Hadjicharalambous, Benoit T. Roux, Eszter Csomor, Carol A. Feghali-Bostwick, Lynne A. Murray, Deborah L. Clarke, Mark A. Lindsay

**Affiliations:** 1 Department of Pharmacy and Pharmacology, University of Bath, Claverton Down, Bath, BA2 7AY United Kingdom; 2 MedImmune, Milstein Building, Granta Park, Cambridge, CB21 6GH United Kingdom; 3 Division of Rheumatology and Immunology, Department of Medicine, Medical University of South Carolina, Charleston, USA; 4 RIA IMED Biotech Unit, AstraZeneca, Gothenberg, Sweden; 5 Present Address: Boehringer Ingelheim Ltd, Ellesfield Avenue, Bracknell, Berkshire RG12 8YS United Kingdom

## Abstract

Phenotypic changes in lung fibroblasts are believed to contribute to the development of Idiopathic Pulmonary Fibrosis (IPF), a progressive and fatal lung disease. Long intergenic non-coding RNAs (lincRNAs) have been identified as novel regulators of gene expression and protein activity. In non-stimulated cells, we observed reduced proliferation and inflammation but no difference in the fibrotic response of IPF fibroblasts. These functional changes in non-stimulated cells were associated with changes in the expression of the histone marks, H3K4me1, H3K4me3 and H3K27ac indicating a possible involvement of epigenetics. Following activation with TGF-β1 and IL-1β, we demonstrated an increased fibrotic but reduced inflammatory response in IPF fibroblasts. There was no significant difference in proliferation following PDGF exposure. The lincRNAs, LINC00960 and LINC01140 were upregulated in IPF fibroblasts. Knockdown studies showed that LINC00960 and LINC01140 were positive regulators of proliferation in both control and IPF fibroblasts but had no effect upon the fibrotic response. Knockdown of LINC01140 but not LINC00960 increased the inflammatory response, which was greater in IPF compared to control fibroblasts. Overall, these studies demonstrate for the first time that lincRNAs are important regulators of proliferation and inflammation in human lung fibroblasts and that these might mediate the reduced inflammatory response observed in IPF-derived fibroblasts.

## Introduction

Idiopathic pulmonary fibrosis (IPF) is a fatal progressive chronic disease characterised by scar tissue accumulation in the lungs leading to impaired gas exchange and restricted ventilation^1–3^. The etiology and pathogenesis of the disease are still unclear, although recent research has indicated that persistent epithelial injury and/or exposure to pathogens, leads to the secretion of fibrotic, proliferative and inflammatory mediators such as TGF-β1^4^, PDGF^5^ and IL-1β^6^ . These are then thought to act upon surrounding fibroblasts, to induce an exaggerated wound healing response that contributes towards the development and progression of IPF^1^.

Comparison of the phenotype of lung fibroblasts derived from IPF patients with those from non-fibrotic patients has shown that these exhibit multiple differences including reduced apoptosis^7,8^ and diminished capacity to synthesis cyclooxygenase 2 and prostaglandin E2^9^. Differences have also been observed in proliferation and release of fibrotic components, although these have resulted in contradictory observations^10–13^. Attempts to understand these persistent phenotypic changes at the epigenetic level have shown differences in the pattern of DNA methylation^14,15^. However, although there are reports of histone changes associated with individual genes linked to IPF^16^, there has been no attempt to determine if there are genome wide changes in the profile of histone modifications.

High-throughput sequencing indicates that much of the human genome is transcribed into non-coding RNAs (ncRNAs). The majority of ncRNAs (>90%) are involved in house-keeping activities such as translation (ribosomal RNA), splicing (short nuclear RNAs) and post-transcriptional RNA modifications (short nucleolar RNA) whilst the others are broadly classified as either short ncRNAs (<200 nt (nucleotides)) or long ncRNAs (lncRNAs) (>200 nt). The microRNA family of short ncRNAs are the best characterised and are known to induce mRNA degradation or block messenger RNA (mRNA) translation via the RNA interference pathway^17^. In contrast, little is known about lncRNAs which are commonly divided into three groups: long intergenic non-coding RNAs (lincRNAs) that are located between protein-coding genes, antisense that are transcribed across protein coding genes on the reverse strand and pseudogenes, that are non-translated versions of protein coding genes^18,19^. In most cases, it is believed that the actions of lncRNAs are mediated through domains that interact with proteins, acting as scaffolds or to modulate their activity^20,21^. At the present time, a number of miRNAs have been implicated in the regulation of fibroblast function and in the development of IPF including let-7d^22^, miR-17~92^23^, miR-101^24^ and miR-155^25^. In contrast, although there is accumulating evidence to indicate that lncRNAs are important regulators of biological response^18,19^, little is known regarding the role of lncRNAs in lung fibroblast function or IPF.

In this report, we have demonstrated differences in the functional responses between fibroblasts derived from control and IPF lungs. These are reflected by changes in H3K4me1, a histone epigenetic marker of primed genes and enhancers^26,27^ and up-regulation of two lincRNAs, LINC00960 and LINC01140 in IPF fibroblasts. Functional analysis has shown that the both LINC00960 and LINC01140 are required for proliferation and that LINC01140 is a negative regulator of the inflammatory response. Given that LINC01140 is upregulated in both IPF fibroblasts and lung biopsies, our data suggest that LINC01140 mediates the reduced inflammatory response in IPF fibroblasts.

## Results

Our initial aim was to determine whether there were significant differences in the phenotypic responses of lung fibroblasts derived from control lung and IPF patients and to examine the potential role of lincRNAs in these variations. We selected high-throughput approaches to measure the time- and concentration dependency of their TGF-β1-induced fibrotic response, PDGF-induced proliferation and IL-1β-stimulated inflammatory response.

### Comparison of the TGFβ1-stimulated PAI-1 release from control and IPF lung fibroblasts

TGF-β1-induced activation of lung fibroblasts triggers the expression of PAI-1 (also known as Serpin E1), a protein known as an important regulator of fibrinolysis and wound healing and therefore implicated in the process of fibrosis^28^. To assess potential difference in the fibrotic response in control and IPF fibroblasts we examined the time- and concentration-dependent release of PAI-1 release in response to TGF-β1.

**Figure 1:**
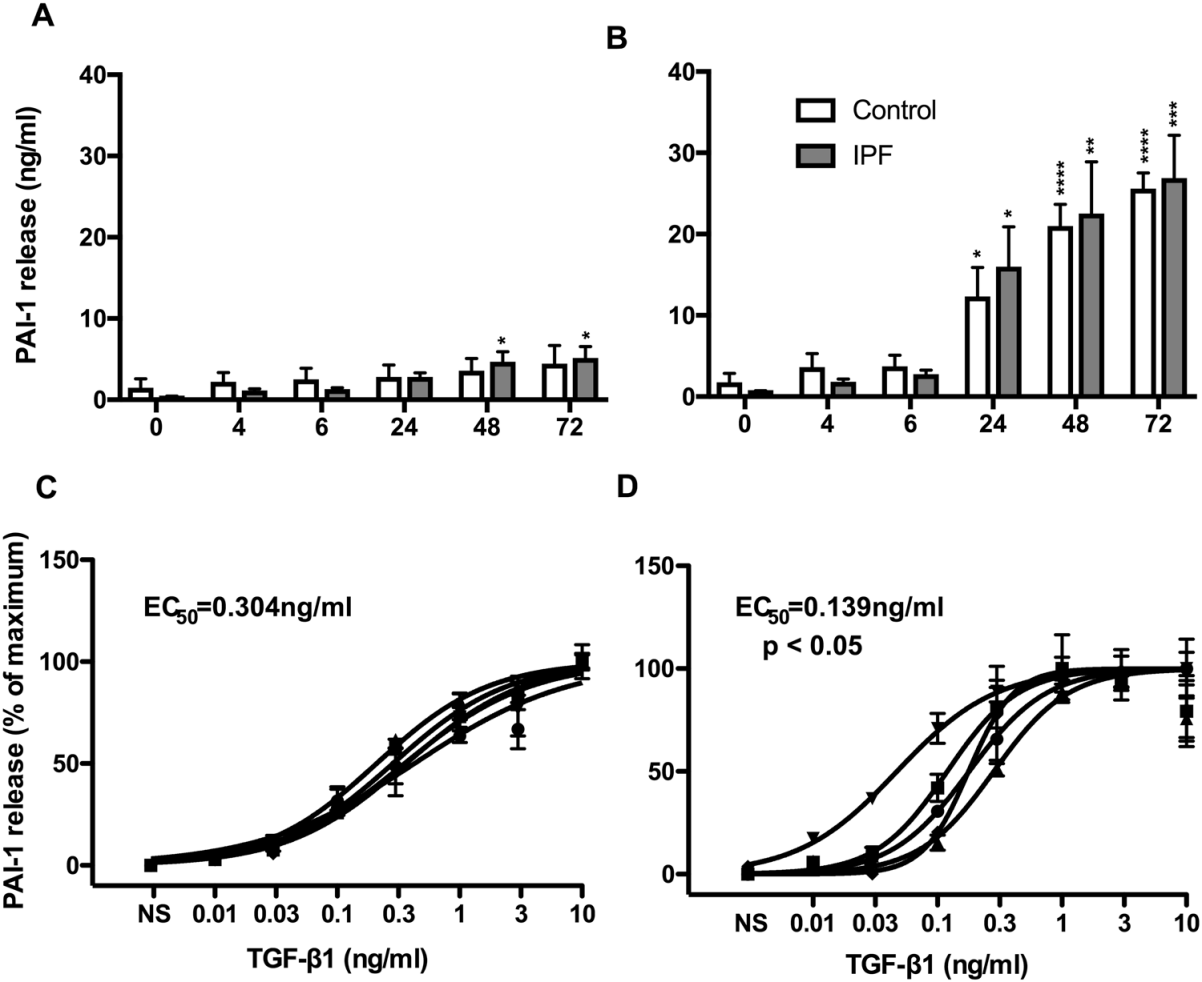
IPF lung fibroblasts showed increased sensitivity to TGF-β1-stimulated PAI-1 release. Time course of PAI-1 release from non-stimulated (**A**) and TGF-β1-stimulated (**B**) fibroblasts derived from control (white) and IPF (grey) patients. PAI-1 release from control (**C**) and IPF (**D**) fibroblasts at 72 h following exposure to the indicated TGF-β1 concentrations. Data represents the mean +/− SEM of five individuals. Statistical significance was performed using 1-way analysis of variance (ANOVA) with a Dunnett’s test for time courses (**A**,**B**) where *p < 0.05, **p < 0.01, ***p < 0.001 and ***p < 0.0001. The logEC_50_ for each individual was determined in GraphPad Prism and comparison between control and IPF groups was performed using a unpaired t-test. The EC_50_ was calculated from the mean logEC50 values.

Initial studies showed a time-dependent release of PAI-1 from non-stimulated IPF cells, which reached significance at 48 hours (h) and 72 h (Fig. 1A). However, comparison between control (4.4 ± 2.2 ng/ml) and IPF (5.1 ± 1.4 ng/ml) at 72 h showed no significant difference. Exposure to 3 ng/ml of TGF-β1 produced a comparable time dependent increase in PAI-1 release from control and IPF fibroblasts, that was significant at 24 h and continued to increase at 48 h and 72 h (Fig. 1B). There was no significant difference between the control and IPF fibroblasts at 72 h with absolute values of 25.3 ± 2.0 ng/ml and 26.6 ± 5.3 ng/ml, respectively.

To examine the concentration-dependent response, cells were incubated for 72 h with 0.01 to 10 ng/ml TGF-β1 and compared with time matched non-stimulated cells. As expected, TGF-β1 induced a concentration-dependent increase in PAI-1 release in both control (Fig. 1C) and IPF (Fig. 1D) fibroblasts that plateaued at ~10 ng/ml and ~3 ng/ml, respectively. The baseline expression in non-stimulated control and IPF fibroblasts was 2.7 ng/ml and 3.4 ng/ml. Comparison of the mean logEC_50_ between the control (319 pg/ml) and IPF (139 pg/ml) individuals showed a significant difference (p < 0.05; unpaired T-test). In relation to PAI-1 release, these studies show IPF fibroblasts are more fibrotic since they demonstrate increased sensitivity to TGF-β1 activation.

### Comparison of PDGF-AB-stimulated proliferation in control and IPF lung fibroblasts

**Figure 2:**
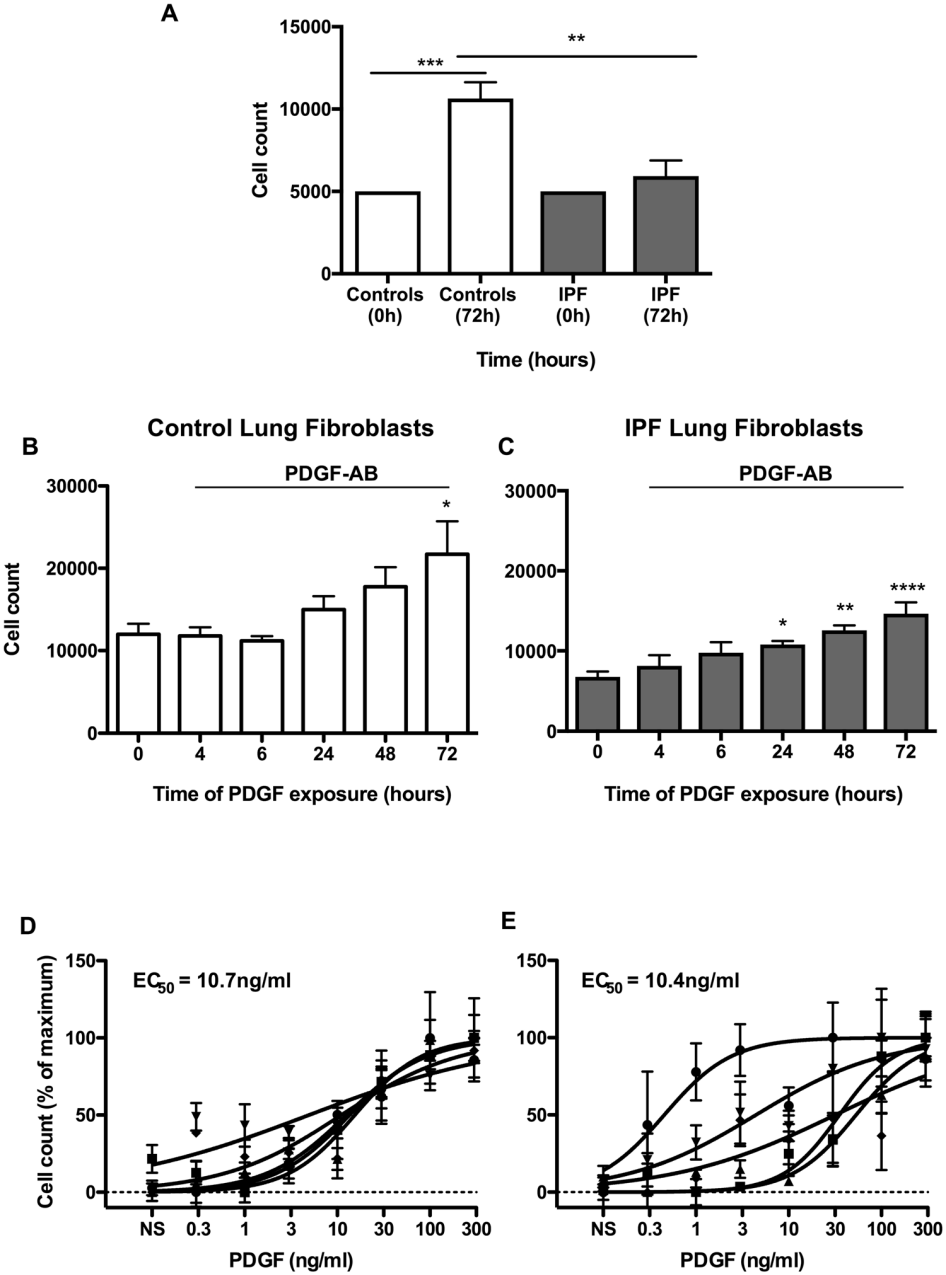
Non-stimulated IPF lung fibroblasts show a reduced proliferative response. Proliferation in non-stimulated control (white) and IPF fibroblasts (grey) was measured 72 h using cell count (**A**). Time course of proliferation in PDGF-stimulated (from control (**B**) and IPF (**C**) patients). Proliferation in control (**D**) and IPF (**E**) fibroblasts at 72 h following exposure to the indicated PDGF concentrations. Data represents the mean +/− SEM of five individuals. Statistical significance was performed using 1-way analysis of variance (ANOVA) with a Dunnett’s or Tukey’s test for time courses (**A**–**C**) where *p < 0.05, **p < 0.01 and ****p < 0.0001. The logEC_50_ for each individual was determined in GraphPad Prism and comparison between control and IPF groups was performed using a unpaired t-test. The EC_50_ was calculated from the mean logEC50 values.

PDGF-AB is a potent mitogen that has been previously shown to stimulate the proliferation of lung fibroblasts^29^. To assess the proliferative response in control and IPF fibroblasts, we investigated the time- and concentration-dependent effect of PDGF-AB on the fibroblast cell number. Initial examination of the proliferation in non-stimulated fibroblasts showed increased proliferation in control versus IPF at 72 h (Fig. 2A). On top of the baseline increases demonstrated in Fig. 2A, exposure to 100 ng/ml PDGF-AB was demonstrated to induce an additional time dependent proliferation in both control and IPF fibroblasts, with an increase 1.8 fold and 2.1 fold at 72 h, respectively (Fig. 2B,C).

Following exposure to recombinant human PDGF-AB (0.3–300 ng/ml), we observed a concentration-dependent increase in proliferation at 72 h, although we observed wide variation in the response of IPF fibroblasts (Fig. 2E) compared with controls (Fig. 2D). Comparison of the mean EC_50_ values showed no significant difference between control and IPF fibroblasts at 10.7 ng/ml and 10.4 ng/ml, respectively. These results indicated that there was a significant small increase in proliferation in non-stimulated control fibroblasts versus IPF although there was no difference in the PDGF-stimulated responses.

### Comparison of IL-1β-stimulated IL-6 release in control and IPF lung fibroblasts

The pro-inflammatory cytokine IL-1β has been shown to potently induce IL-6 release from various cell types including fibroblasts^30,31^. To assess potential differences in the inflammatory response, we examined the time- and concentration-dependent IL-1β induced IL-6 release from control and IPF lung fibroblasts.

**Figure 3:**
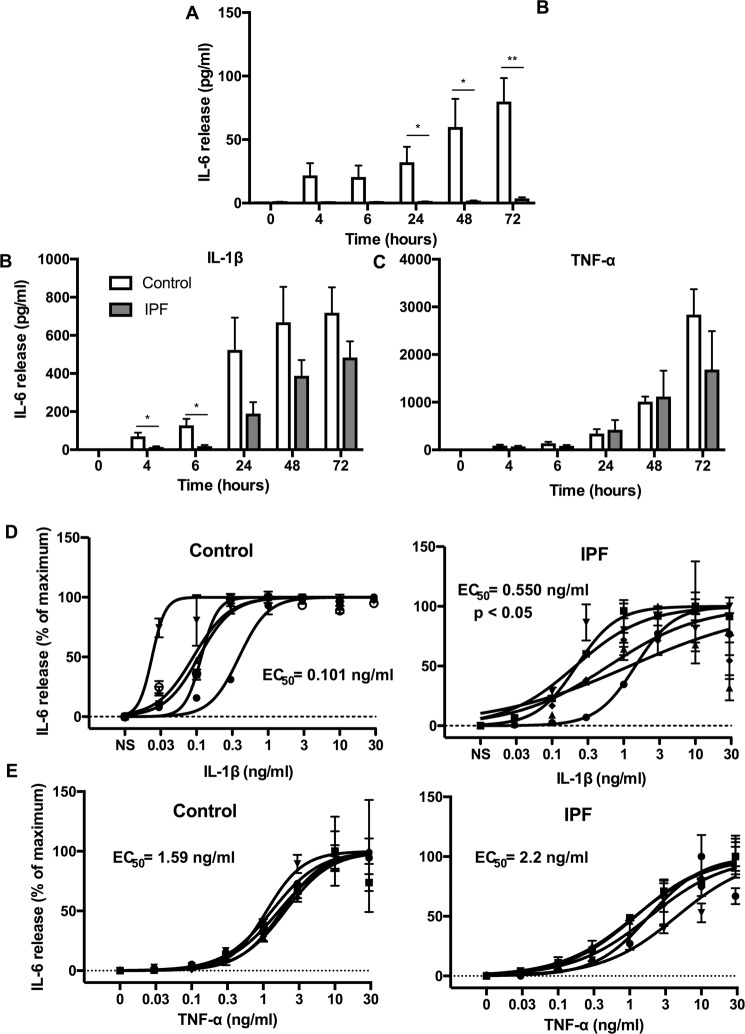
IPF lung fibroblasts show a reduced IL-1β-stimulated inflammatory response. Time course of IL-6 release from non-stimulated (**A**) and IL-1β-stimulated (**B**,**D**) and TNFα-stimulated (**C**,**E**) fibroblasts derived from control (white) and IPF (grey) patients. IL-6 release from control and IPF fibroblasts at 24 h following exposure to the indicated IL-1β (**D**) and TNF-α (**E**) concentrations. Data represents the mean +/− SEM of five individuals. Statistical significance was performed using an unpaired t-test where *p < 0.05 and **p < 0.01. The logEC_50_ for each individual was determined in GraphPad Prism and comparison between control and IPF groups was performed using an unpaired t-test. The EC_50_ was calculated from the mean logEC50 values.

Interestingly, we observed a time dependent IL-6 release in non-stimulated fibroblasts, which was significantly increased in control (67.1 ± 19.8 pg/ml) versus IPF (3.7 ± 0.9 pg/ml) fibroblasts at 24 h, 48 h and 72 h (Fig. 3A). Exposure to 3 ng/ml IL-1β induced a comparable time-dependent release of IL-6 from control and IPF fibroblasts, which was initially detected at 4 h and continued to increase over the 72 h period, with a maximum of 723 ± 134 pg/ml and 483 ± 85 pg/ml, respectively (Fig. 3B). There was no significant difference in the maximal values between control and IPF at 72 h although there was a significant increase in control fibroblasts at 4 h and 6 h (Fig. 3B).

To examine the concentration-dependency, cultured cells were incubated for 72 h with increasing concentrations of IL-1β ranging from 0.03 to 30 ng/ml (Figure D). Comparison of the logEC_50_ values showed a significant reduction (p = 0.0234) between control (EC_50_ = 101 pg/ml) and IPF (EC_50_ = 550 pg/ml) fibroblasts. Based upon IL-6 release, this data would indicate that IPF fibroblasts are less inflammatory than control cells as they demonstrated both reduced sensitivity to IL-1β and a lower basal release of IL-6.

To assess whether the response to IL-1β in IPF fibroblasts is generic, we also examined the response to another pro-inflammatory mediator, TNF-α^30,31^. Exposure to 10 ng/ml TNF-α induced a comparable time-dependent release of IL-6 from control and IPF fibroblasts, which continued to increase over the 72 h period (Fig. 3C). Once again there was no significant difference in the maximal values between control and IPF at 72 h. In contrast to IL-1β, examination of the concentration-dependency, showed no difference in the TNF-α-induced IL-6 response between control (EC_50_ = 1.6 ng/ml) and IPF (EC_50_ = 2.2 ng/ml) fibroblasts (Fig. 3E).

### Phenotypic differences between control and IPF fibroblasts are reflected at the epigenetic level

**Figure 4:**
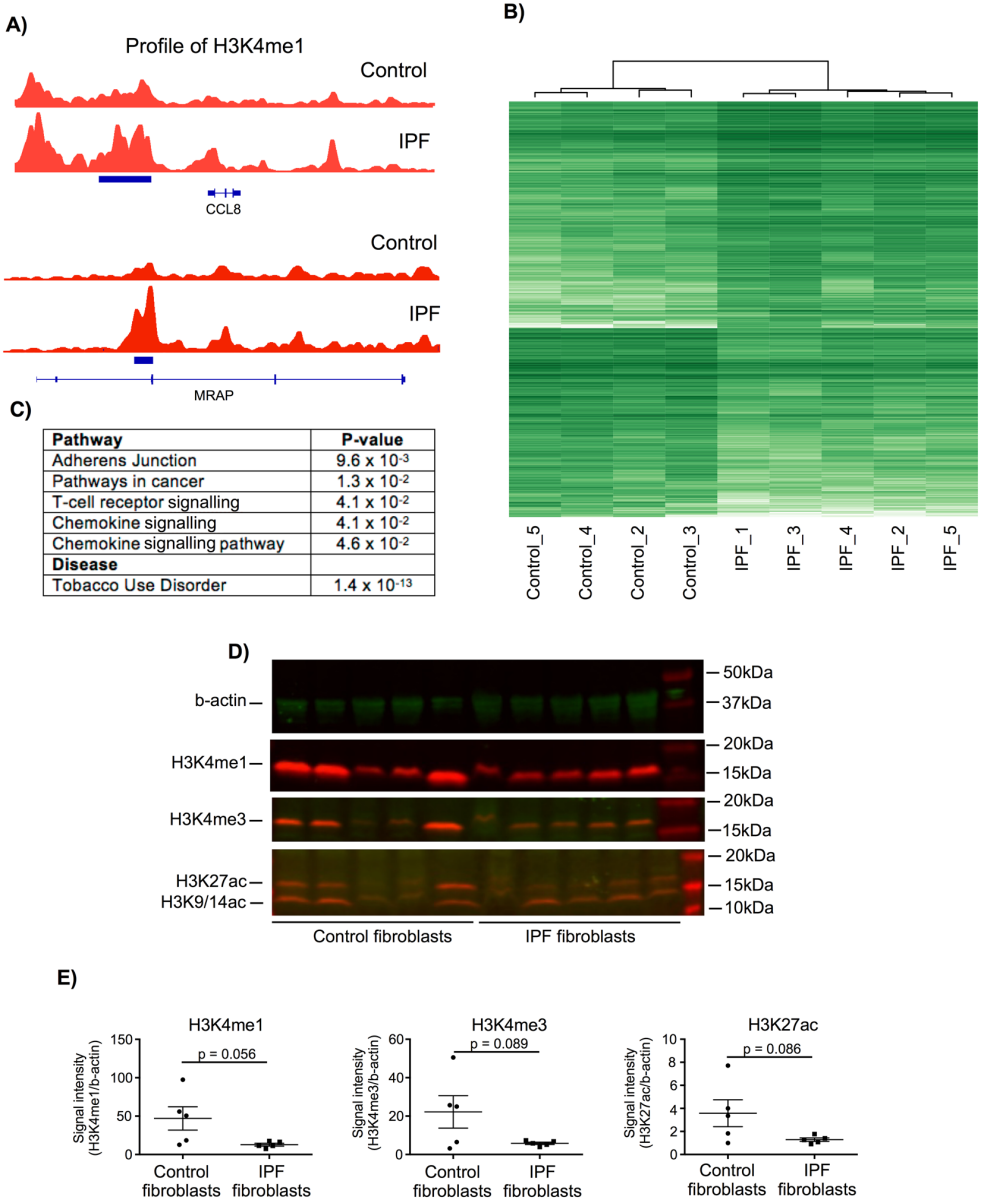
Differential expression of the histone epigenetic mark between control and IPF fibroblasts. ChIP sequencing was employed to examine the differential expression of H3K4me1, a marker of primed promoter and enhancer regions. (**A**) examples of the H3K4me1 regions associated with CCL8 and MRAP, (**B**) unsupervised hierarchical clustering was calculated within the DiffBind programme and (**C**) pathways analysis of H3K4me1 associated genes was undertaken using DAVID. Total expression of the histone marks H3K4me1, H3K4me3 and H3K27ac in control and IPF fibroblasts were measured by Western blotting (**D**) and then quantified by densitometry using b-actin as an internal loading control (**E**) where data is the mean +/− SEM of 5 donors and statistical difference was examined using an unpaired T-test. The section D contains cropped gels and the original uncropped gels can be viewed in Supplemental Fig. 1.

Having demonstrated differentially functional responses between control and IPF fibroblasts, we examined whether these differences were reflected at the epigenetic level. To this end we examined the histone modification H3K4me1, a marker that has previously been associated with primed promoter and enhancer regions^26,27^. Comparison between non-stimulated control and IPF fibroblasts identified 462 regions of differential expression (Supplemental Dataset 1). As examples, we have included the profiles of the H3K4me1 peaks associated with CCL8 and MRAP (Fig. 4A). Unsupervised hierarchical clustering showed a clear difference (separation) of the control and IPF samples (Fig. 4B) indicating that the phenotypic differences are indeed reflected at the epigenetic level. Pathway analysis of the genes in which these regions overlapped or which were closest (Supplemental Dataset 1) showed that these were associated with tight junctions, cancer and inflammation (Fig. 4C). Interestingly, these genes were also strongly associated with tobacco user disorder (1.4 × 10^−13^) despite no history of smoking in either the control or IPF patients. To examine further the potential role of epigenetics changes, we have employed Western blotting to compare the levels of H3K4me1, H3K4me3 and H3K27ac in the five control and IPF patient samples (Fig. 4D,E – full gel images available in Supplemental Dataset 2). As a result of the variation in the control samples, these narrowly failed to reach significance. However, this shows a trend towards reduced expression of H3K4me1, H3K4me3 and H3K27ac in IPF fibroblasts and might explain the reduced inflammatory response.

### Differential long non-coding RNA expression between control and IPF fibroblasts

**Figure 5:**
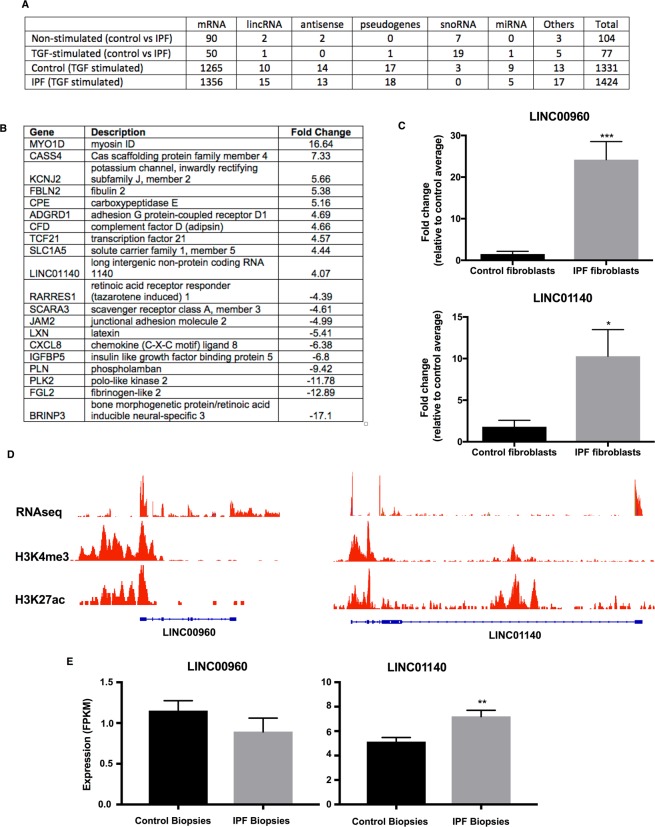
Transcriptome analysis shows differential expression of long intergenic RNA between control and IPF fibroblasts. (**A**) The differential expression of various classes of genes was examined in 5 control and 5 IPF fibroblasts samples in the presence and absence of TGF-β1 stimulated at 24 h. (**B**) Table showing the genes with highest fold changes in non-stimulated fibroblasts at 24 h. (**C**) The differential expression of the two lincRNAs, LINC00960 and LINC01140 was confirmed by qRT-PCR (n = 5). (**D**) LINC00960 and LINC01140 expression was confirmed by comparison with RNA sequencing data and the epigenetic marks associated with H3K4me3 (active promoters) and H3K27ac (active transcription). (**E**) Expression of LINC00960 and LINC01140 in the lung biopsies of control (n = 19) and IPF patients (n = 20). Data in (**D**,**E)** are the mean +/− SEM and statistical significance was performed using an unpaired t-test where *p < 0.05, **p < 0.01 and ***p < 0.001.

Having demonstrated differentially functional responses between control and IPF fibroblasts, we wondered whether these might be related to long non-coding RNAs (lncRNAs). To this end, we compared the expression profile in control and IPF lung fibroblasts in non-stimulated cells and those exposed to 3 ng/ml TGF-β1 for 24 h, using the Affymetrix GeneChip™ Human Transcriptome Arrays 2.0 (Supplemental Dataset 3). TGF-β1 exposure resulted in widespread and shared changes in gene expression in both control (1331 genes including 10 lincRNAs and 14 antisense: Supplemental Dataset 4) and IPF fibroblasts (1424 genes including 15 lincRNAs and 13 antisense: Supplemental Dataset 5) (Fig. 5A,B). We were unable to show a correlation between the histone peaks and those mRNAs and lincRNAs that were significantly changed in unstimulated control versus IPF patients. As might be expected, pathway analysis (DAVID Bioinformatics) showed that the highest hit for the up-regulated genes were extracellular matrix (9.9 × 10^−15^). Amongst this group, PAI-1 (Serpin E1) gave one of the highest fold changes in both the control (44-fold) and IPF fibroblasts (31-fold). Comparison of the lists of genes that were changed following TGF-β1 exposure identified only 77 that were differentially expressed between control and IPF, which includes a single lincRNA (LOC100507516) whose expression was reduced in IPF (Supplemental Dataset 6).

Given the emerging evidence to suggest that lincRNAs are novel regulators of biological responses, we decided to examine their potential role in mediating the phenotypic changes in IPF fibroblasts. To identify those lncRNAs that might regulate the fibrotic, proliferative and inflammatory response, we compared the profile of gene expression in non-stimulated fibroblasts. This identified differential expression of 104 genes including 2 lincRNAs (LINC00960 and LINC01140) that were increased in IPF (Supplemental Dataset 7). Interestingly, there was also a general down-regulation of small nucleolar RNAs (snoRNAs), that are commonly associated with splicing, as well as changes in a number of inflammatory genes including CXCL8 down-regulation and CXCL11 up-regulation (Supplemental Dataset 7).

In subsequent functional studies, we focused upon the role of the lincRNAs, LINC00960 and LINC01140. Initial qRT-PCR analysis confirmed their upregulation in IPF fibroblasts (Fig. 5C) and their existence was confirmed following analysis of our own RNA sequencing data (GSE121241) and ENCODE ChIP-seq data of two epigenetic markers of active promoters (H3K4me3: ENCFF626WKG) and transcription (H3K27ac: ENCFF637KNN) (Fig. 5D). To ascertain whether these are also upregulated in IPF lung *in situ*, we analysed RNA sequencing data of biopsy samples obtained from control (n = 19) and IPF (n = 20) lungs^32^ (Supplemental Dataset 8) and showed significant upregulation of LINC01140 but not LINC00960 (Fig. 5E).

### Long intergenic non-coding RNAs and the regulation TGF-β1-stimulated PAI-1 release from control and IPF lung fibroblasts

**Figure 6:**
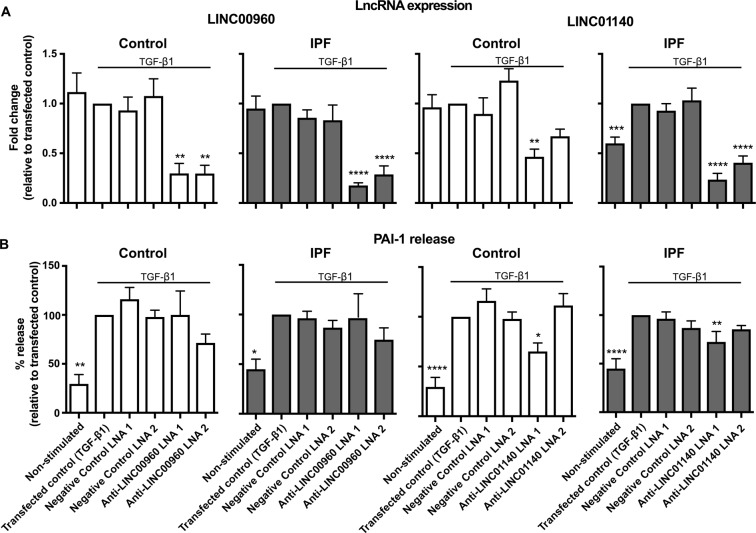
LincRNAs and the regulation of TGF-β1-stimulated PAI-1 release. Control and IPF fibroblasts were transfected with LNA antisense sequences against LINC00960, LINC01140 or scrambled controls overnight. Cell were then stimulated with TGF-β1 for 72 h prior to (**A**) isolation of mRNA and measurement of LINC00960 or LINC01140 by qRT-PCR or (**B**) measurement of supernatant PAI-1 by ELISA. Data represents the mean +/− SEM of five control or IPF individuals. Statistical significance was performed using the repeat measures 1-way analysis of variance (ANOVA) with a Dunnett’s test where *p < 0.05, **p < 0.01, ***p < 0.001 and ****p < 0.0001.

To investigate the function of LINC00960 and LINC01140, we identified 2 locked nucleic acid based (LNA) antisense sequences against each lincRNA that produced 50–85% knockdown following overnight transfection into fibroblasts and stimulation with TGF-β1 for 24 h (Fig. 6A). Following exposure to TGF-β1, we observed a significant increase in PAI-1 release from both control and IPF fibroblasts (Fig. 6B), although the magnitude of this response was smaller than that observed in non-transfected cells (Fig. 1). Knockdown of LINC00960 had no effect upon PAI-1 release from both control and IPF fibroblasts. Although one LNA antisense against LINC01140 caused a significant reduction in PAI-1 release from control fibroblasts (Fig. 6B), taken as a whole, it appears that LINC01140 also does not regulate PAI-1 release. Two scrambled negative LNA controls had no effect on either lincRNA expression or PAI-1 release. These results indicate that neither LINC00960 nor LINC01140 are required for the PAI-1 fibrotic response in control and IPF fibroblasts.

### Long intergenic non-coding RNAs and the regulation of PDGF-stimulated proliferation in lung fibroblasts

**Figure 7:**
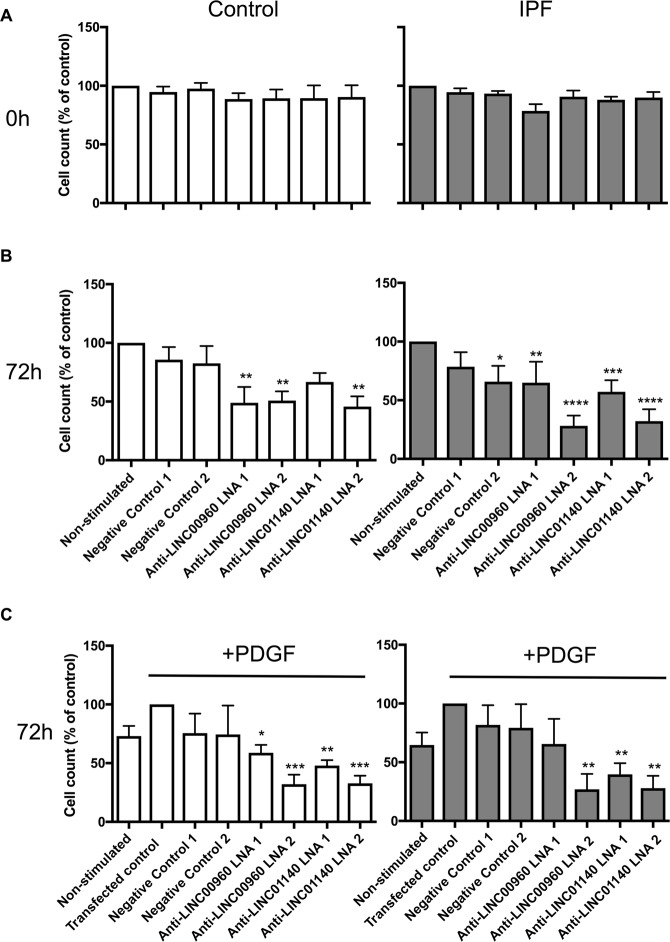
LincRNAs and the regulation of the PDGF-stimulated proliferation. Control and IPF fibroblasts were transfected with LNA antisense sequences against LINC00960, LINC01140 or scrambled controls overnight. The cell number was determined at 0 h (**A**) or in non-stimulated (**B**) and PDGF-stimulated (**C**) samples at 72 h. Data represents the mean +/− SEM of five control or IPF individuals. Statistical significance was performed using the repeat measures 1-way analysis of variance (ANOVA) with a Dunnett’s test where *p < 0.05, **p < 0.01, ***p < 0.001 and ****p < 0.0001.

Following overnight transfection with LNA antisense to LINC00960, LINC01140 and the 2 negative controls, we observed no effect upon cell count, indicating that this procedure had no immediate action upon cell viability (Fig. 7A). Following 72 h culture, knockdown of LINC00960 and LINC01140 caused a significant reduction in proliferation in both non-stimulated (Fig. 7B) and PDGF-stimulated (Fig. 7C), which was seen in both control and IPF fibroblasts. Generally, no reduction was observed with the negative LNA controls. These results indicate that LINC00960 and LINC01140 are positive regulators of fibroblast proliferation.

### Long intergenic non-coding RNAs and the regulation of IL-1β-stimulated IL-6 release in control and IPF lung fibroblasts

**Figure 8:**
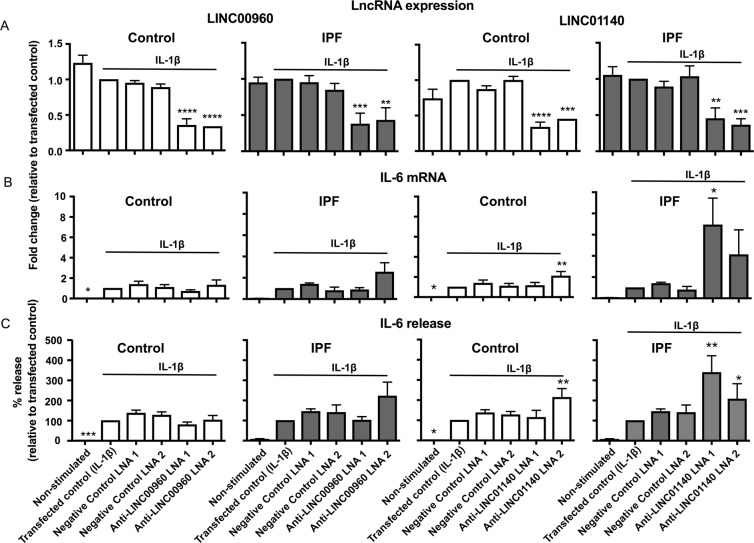
LincRNAs and the regulation of the IL-1β-stimulated IL-6 release. Control and IPF fibroblasts were transfected with LNA antisense sequences against LINC00960, LINC01140 or scrambled control overnight. Cell were then stimulated with IL-1β for 24 h prior to isolation of RNA and measurement of LINC00960 or LINC01140 (**A**) and IL-6 (**B**) by qRT-PCR or (**C**) measure of released IL-6 by ELISA. Data represents the mean +/− SEM of five control or IPF individuals. Statistical significance was performed using the repeat measures 1-way analysis of variance (ANOVA) with a Dunnett’s test where *p < 0.05, **p < 0.01, ***p < 0.001 and ****p < 0.0001.

We once again demonstrated LNA mediated knockdown of LINC00960 and LINC01140 in both control and IPF fibroblasts following overnight transfection and 24 h exposure to IL-1β (Fig. 8A). LINC000960 knockdown had no effect upon either IL-1β-induced IL-6 expression (Fig. 8B) or IL-6 release (Fig. 8C) from either control or IPF fibroblasts. In contrast, LINC01140 knockdown in IPF fibroblasts resulted in a ~4–7 fold increase in IL-6 mRNA expression (Fig. 8B) and ~2–3 fold increase in IL-6 release (Fig. 8C) although the variability between patients meant that this was only significant with LNA1. A much smaller ~2 fold increase in IL-6 mRNA and protein was also seen with LNA2 against LINC01140 in control fibroblasts (Fig. 8B,C). These observations indicate that LINC01140 is a negative regulator of the IL-1β-stimulated IL-6 release, particularly in IPF fibroblasts. Interestingly, the increased expression of LINC01140 in IPF fibroblasts might explain their reduced response to IL-1β compared to control fibroblasts (Fig. 3).

## Discussion

In this report, we have for the first time investigated the role of lincRNAs in the regulation of lung fibroblast function and whether changes in their expression might be involved in the development of IPF. In our initial studies, we employed high throughput assays to undertake detailed examination of the time and concentration responses of control and IPF fibroblasts, examining phenotypes associated with IPF^1,3^. These included TGF-β1-stimuated PAI-1 release as a marker of the fibrotic response^33,34^, PDGF-induced changes in cell numbers as a model of proliferation^35,36^ and IL-1β-stimulated IL-6 release as a marker of inflammation^37^.

As previously reported when using collagen release as a measure of fibrosis, we observed no difference in the magnitude of PAI-1 release from control and IPF fibroblast in the absence and presence of a maximally effective TGF-β1 concentration^13^. In contrast, the concentration response curves showed a significant leftward shift in IPF fibroblasts, indicating that these had increased sensitivity to TGF-β1.

In the absence of PDGF, we observed a reduction in the proliferation of IPF fibroblasts. Following exposure to PDGF, the small increases and the variability of the response between individual fibroblasts samples, meant there was no significant difference in the magnitude or concentration dependency of the proliferation. This variability in the PDGF-induced proliferative response had previously been observed by Jordana *et al*.^10^.

In general, IL-1β is considered to be a potent pro-inflammatory cytokine that can induce the release of multiple pro-inflammatory mediators, including IL-6^38^. Unlike TGF-β1 and PDGF, the role of IL-1β in the development of IPF is yet to be established although there is a report of showing increased levels in bronchoalveolar lavage^39^. In addition, there are conflicting reports as to whether IL-1β elicits profibrotic or antifibrotic activities^40,41^. IL-1β was previously shown to drive IL-6 expression in orbital and synovial fibroblasts *in vitro*^42^, although nothing is known regarding its effect on lung fibroblasts. Significantly, measurement of IL-6 indicated that non-stimulated IPF fibroblasts were less inflammatory and showed a reduced sensitivity to IL-1β stimulation. In contrast, examination of the IL-6 release in response to TNF-α showed no difference between control and IPF fibroblasts indicating that reduced sensitivity might be specific to IL-1β. In support of these phenotypic observations, a recent meta-analysis of four previous microarrays studies in control and IPF fibroblasts which showed repression of inflammation and immune pathways^43^. Interestingly, this reduced inflammation in non-stimulated and IL-1β-stimulated IPF fibroblasts may explain the lack of efficacy observed when corticosteroids and immunosuppressants have been examined as therapeutic options for the treatment of IPF^1,3^.

Having demonstrated significant differences in fibrotic and inflammatory responses between control and IPF fibroblasts, we then proceeded to show that this was also reflected at the epigenetic level. To date, the only genome wide epigenetic studies in IPF have examined the changes in the profile of DNA methylation^14,15^, with none having examined histone modifications. We therefore measured the distribution of H3K4me1, a marker for priming at promoter and enhancer regions that is thought to work through the recruitment of multiple chromatin-remodelling complexes^26,27,44^. Significantly, we identified multiple regions demonstrating differential expression of H3K4me1, with unsupervised hierarchical clustering showing a clear separation between control and IPF fibroblasts. In addition, although the patients variation meant that we failed to reach significance, we have shown a trend towards reduced expression of H3K4me1, H3K4me3 and H3K27ac in IPF fibroblasts and speculate that this might to linked to phenotypic changes such as the reduced inflammatory response. Although there have been a number of reports showing changes is histone marks localised to specific genes implicated in IPF^16^, this is the first evidence to show genome-wide changes in histone modifications and that targeting the acetylation of H3K4 and other histones might provide a novel therapeutic strategy. Indeed, this latter contention is supported by studies into Brd4, a bromodomain containing protein that can bind to acetylated histones and act as a scaffold to attract components of the transcriptional machinery. Thus, inhibitors of Brd4 have been shown to attenuate migration, proliferation and IL6 release in isolated fibroblasts and inhibit fibrosis in a bleomycin-induced model of flung fibrosis^44,45^.

At the present time, little is known regarding the role of lincRNA either in the regulation of fibroblast function or whether changes in their expression are associated with the phenotypic changes associated with IPF. The exception is studies showing that H19^46,47^ and pulmonary fibrosis associated lncRNA (PFAL)^48^ contribute to lung fibrosis by acting as sponges (and therefore inhibitors) for miRNAs. Using microarray analysis and qRT-PCR, we identified 2 lincRNAs, LINC00960 and LINC01140 that were up-regulated in IPF compared to control fibroblasts. Given that isolated and cultured fibroblasts might not reflect the situation in the whole lung, we were able to analyse historical RNA sequencing data obtained from lung biopsies^32^ and confirm up-regulation of LINC01140 but not LINC00960. Knockdown studies indicated that neither appeared to regulate TGF-β1-induced PAI-1, although it is not possible to eliminate the possibility that these lincRNAs might regulate another aspect of the fibrotic response. In contrast, knockdown studies indicated that both LINC00960 and LINC01140 were required for proliferation. Interestingly, these knockdown studies appeared to contradict the results in non-stimulated cells which showed increased proliferation in control fibroblasts despite reduced levels of LINC00960 and LINC01140. The reason for this discrepancy is unknown, although we speculated that other pathways, rather than LINC00960 and LINC00960, might play a dominant role in regulating the increased proliferation in non-stimulated control fibroblasts. It might also be argued that measurement of cell number is not the optimal approach and that lincRNA knockdown may have impacted upon cell viability rather than decreased proliferation. However, we believe that this is unlikely since we observed no significant effect of lincRNA knockdown upon TGF-β1-induced PAI-1 release and increased IL6 release in response to IL-1β exposure, when decreased cell viability might have been expected to also reduce these responses. Examination of the inflammatory response showed that LINC01140 was a negative regulator of IL-1β-induced IL-6 release. In the latter case, there was a greater elevation in IL-6 release from IPF fibroblasts following LINC001140 knockdown, possibly reflecting the increased expression of LINC01140 in IPF versus control fibroblasts. This increased LINC01140 levels in IPF fibroblast might also explain the absence of IL-6 release in non-stimulated IPF fibroblasts compared with controls and the shift in the concentration response curve in response to IL-1β exposure. Although the mechanism of action is yet to be determined, negative regulation of the inflammatory response is commonly observed with both lncRNAs and miRNAs including interleukin-7-antisense (IL7-AS)^49^ and miR-146a^50^. Of relevance, we have recently shown that increased inflammatory response is IPF fibroblast might also be related to reduced expression of MIR3142HG/miR-146a^51^.

Overall, this report is the first to demonstrate that phenotypic differences between control and IPF fibroblasts are associated with genome-wide changes in histone modifications and increased expression of the lincRNAs, LINC00960 and LINC01140. Significantly, we also demonstrate that these lincRNAs can regulate fibroblast proliferation and inflammation whilst changes in LINC01140 expression might mediate the reduced inflammatory in IPF fibroblasts. Currently, the mechanisms by which these lincRNAs regulate these responses is unknown. By analogy with proteins and the non-coding RNAs involved in mRNA translation (tRNAs/rRNAs) and splicing (snRNA), it is speculated that the biological actions of lncRNAs are mediated through domains containing conserved sequences, that interact with proteins and/or base pair with RNA/DNA^20,52,53^. With regards to inflammation, immune-modulatory lncRNAs have been shown to bind and regulate the activity of (i) transcription factors such as nuclear factor-κB (NF-κB), signal transducer and activator of transcription 3 (STAT3) and the glucocorticoid receptor (GR), and (ii) chromatin associated proteins including the heterogeneous nuclear ribonucleoproteins (hnRNPs) family and components of the polycomb repressor complex 2 (PRC2)^21,54,55^. However, the identification of these functional domains (conserved sequences) has been hindered by their poor evolutionary conservation, which in contrast to protein coding genes, does not require the maintenance of a conserved open reading frame for optimal translation. Instead, it is thought that lncRNAs conservation is geared towards the maintenance of genomic position (synteny) and short conserved domains (microdomains)^20,52,53^. In addition, it has been suggested that the action of lncRNAs might be mediated through acting as sponges for miRNAs, although this remains a controversial issue^,56,57^. Future studies will examine the mechanism of action of LINC00960 and LINC01140 in fibroblasts responses.

## Methods

### Source and fibroblast cell culture

Control (age = 50 ± 3 y; 3 male and 2 females) and IPF fibroblasts (age = 62 ± 1 y; 3 male and 2 females) were obtained from Professor Carol Ferghali-Bostwick (Medical University of South Carolina, USA) and the Coriell Institute of Medical Research (Camden, New Jersey, USA). Approval was obtained from the Medical Board of the Medical University of South Carolina and the Coriell Institute of Medical Research with patients proving material with informed consent. All methods were performed in accordance with the relevant guidelines and regulations. Neither the control or IPF patients had a history of smoking. Isolation of lung fibroblasts was initiated using explants of minced lung tissue. Fibroblasts were cultured in DMEM (high glucose, pyruvate) growth media (11995-073, ThermoFisher) supplemented with 10% (v/v) FBS (Fetal Bovine Serum) (11550356, ThermoFisher), 1% (v/v) Penicillin-Streptomycin (11548876, ThermoFisher) and 0.1% (v/v) Fungizone (15290-018, ThermoFisher). All cultures were maintained in a 37 °C, 5% (v/v) CO_2_ humidified incubator. Upon reaching approximately 80–90% confluency cells were washed in sterile 1x PBS (Phosphate Buffered Saline) (P5493, Sigma-Aldrich) followed by treatment with StemPro® Accutase® cell detachment solution (11599686, ThermoFisher). All experiments were performed using cells plated at passage 6 to 7.

### Plating and treatment of fibroblasts for pharmacological studies

1 × 10^4^ cells were plated in 96 cell culture wells on day 1 and allowed to adhere overnight. On day 2, cells were serum-deprived by reducing FBS to 0.1% in 200 μl of starvation media. Fibroblasts were then treated with the required concentration of TGF–β1 (recombinant human, expressed in Chinese hamster ovary cell line, R&D systems, 240-B-002/CF), IL-1β (recombinant, expressed in *E*. *coli*, Sigma-Aldrich, I9401-5UG), TNF-α (recombinant, E. coli derived human TNF-alpha protein, R&D systems, 210-TA-005) and PDGF-ΑΒ (recombinant human, *E*. *coli* derived, R&D systems, 222-AB-010) and incubated for the indicated time before supernatants were collected for protein quantification assessment or cell viability assays.

### Preparation and treatment of fibroblasts used in microarray analysis

5 × 10^5^ cells of control and IPF lung fibroblasts were seeded in 25 cm^2^ cell culture flasks (Nunc EasyFlasks, Thermo Fisher Scientific) and medium was replaced until the cells reach near-confluency state. Their growth medium was then replaced with 3 ml starvation medium (0.1% FBS) and left overnight. The following day, the cells were treated with and without 3 ng/ml TGF-β1 for 24 h before all supernatants were collected and cells were lysed.

### Cell Proliferation

Lung fibroblast proliferation was evaluated by measuring cell viability with the Cell Counting Kit-8 (CCK-8, Sigma-Aldrich). Cells (5000) were seeded in a 96-well plate and incubated overnight in 100 μl growth media (10% FBS). The following day, the cells were serum-deprived and treated with the indicated concentration of PDGF-ΑΒ and then incubated for the indicated times. Before the end of each assay, 10 μl of the CCK-8 solution was added into a final volume of 100 μl cultured media and incubated for 2 h before absorbance was measured using a microplate reader (Fluostar Optima, BMG Labtech). The absorbance wavelength was measured at 450 nm and 600 nm which was then subtracted during data analysis.

### Transfection with LNA antisense

On the day of transfection, 5 µL of HiPerFect (Qiagen) was mixed with 200 µL of growth media without antibiotics, serum or antifungals to prepare the transfection mix. LNA Gapmers were added to 200 µL of the transfection mix at a final concentration of 30 nM, placed in 12-well plates and incubated for minimum 10 mins at room temperature. Fibroblasts were then seeded at a density of 5 × 10^5^ cells per well in 200 µL of growth media and incubated with the transfection mixes at 37 °C, 5% (v/v) CO_2_ overnight. The next day, 800 µL of media (0.1% FBS) was added to the wells to dilute out the lipid-LNA complexes and reduce the toxicity of the reaction. The cells were stimulated with either 3 ng/ml of TGF-β1 or 3 ng/ml of IL-1β and incubated for 24 h before harvesting for RNA extraction and ELISA analysis. The same transfection protocol was followed for the cell viability assays (CCK-8 kit) with or without 100 ng/ml of PDGF-AB stimulation for 72 h, however 96 well plates were used and the reaction volume and reagents were reduced accordingly. LNA Gapmer sequences: LNA1 –TCATACTATATGACAG; Negative Control LNA2 – GACGGTAAGTAGGCGA; LINC00960 LNA1 – GGCGTGAGAGTAAAGC; LINC00960 LNA2 – GTGCTTAGGCTTAGAG; LINC01140 LNA1 – TTTAATTGGGCCGTCT; LINC01140 LNA2 – TTGACACGGCTGACTT.

### Measurement of IL-6 and PAI-1 release

Supernatants of cultured lung fibroblasts were collected and used to assess secretion of IL-6 and PAI-1, using the DuoSet ELISA (Enzyme-linked immunosorbent assay, DY206 and DY1786) Development System Kits (R&D Systems Europe, UK) following the manufacturer’s instructions. Samples and standard curve samples were diluted as appropriate in reagent diluent. Absorbance was measured at 450 nm with wavelength correction at 570 nm using a microplate reader (Fluostar Optima, BMG Labtech).

### RNA isolation and quality control

For all samples, total RNA was extracted using the RNeasy kit (Qiagen), included an on-column DNase treatment (Qiagen), according to the manufacturer’s guideline. RNA concentration was determined using the Qubit 2.0 (Life Technologies). RNA quality was measured using the Agilent Bioanalyzer and produced RIN values of >8.0.

### Quantitative PCR validation of lncRNA expression

For quantitative PCR (qPCR), cDNA libraries were prepared from total RNA using the High capacity cDNA RT kit (Applied Biosystems, Life Technologies, No 4368813). Expression of mRNAs and lncRNAs were determined by qPCR using the SYBR® Green PCR mix (Applied Biosystems; primers were obtained from Sigma-Aldrich and are listed in Supplementary Table 1). For analysis, the 2^−(ΔΔCt)^ method was used to determine relative-quantities of individual mRNAs and lncRNAs which were normalized to 18S ribosomal RNA. qRT-PCR primer sequences: 18S – AAACGGCTACCACATCCAAG (Forward), CCTCCAATGGATCCTCGTTA (Reverse); IL-6 – ACTCACCTCTTCAGAACGAATG (Forward), CCATCTTTGGAAGGTTCAGGTTG (Reverse); LINC00960 – TCCAGGCGTCATAACCAACC (Forward), CGGTGCTTAGGCTTAGAGGG (Reverse); LINC01140 – CATCTCATCGGCATGGACCT (Forward), CAAACTGGACTGACTTTCACCA (Reverse).

### Western blot

Whole lysates of control and IPF fibroblasts were harvested in RIPA buffer (Sigma-Aldrich, R0278) supplemented with a protease inhibitor cocktail (Thermo Fisher Scientific, 78430). SDS-PAGE electrophoresis was used to separate proteins using the Mini-PROTEAN® TGX™ Precast Gels (Bio-Rad, 4561085) with Precision Plus Protein™ Dual Xtra Prestained Protein Standards (Bio-Rad, 1610377). Samples were transferred to PVDF membranes using the Trans-Blot® Turbo™ Transfer System (Bio-Rad) and the membranes were blocked for 1 h at room temperature (RT) in blocking buffer (3% BSA in PBS) before they were incubated overnight at 4 °C in a 3% BSA-TTBS (Tris-buffered saline with Tween) blocking buffer with the following antibodies: β-actin (BioLegend, 643802), H3K4me1 (Diagenode, C15410194), H3K4me3 (Diagenode, C15410003) and H3K27ac (Diagenode, C15410196). The membranes were washed 3 times in TTBS for 5 mins and incubated with IRDye® 800CW Goat anti-Mouse (LI-COR, 926-32210) and IRDye® 680RD Donkey anti-Rabbit (LI-COR, 926-68073) for 1 h at RT before they were washed and imaged on the LI-COR Odyssey CLx imaging system.

### Transcriptome analysis of microarray data

The Affymetrix GeneChip™ Command Console Software was used to summarise probe intensity data and to generate a CEL file for each sample. The CEL files were then processed by the Affymetrix Expression Console™ using Robust Multi-chip Analysis (RMA) to generate CHP files (Probe-level summarisation) following the manufacturers manual. The CHP files were then used for Quality Control analysis which generated a full report with the array QC metrics and appropriate algorithm parameters. Using the data of the CHP files, a 3-dimensional Principle Component Analysis (PCA) graph was generated accounting for the majority of variance based on a set of variables PCA1, PCA2 and PCA3 in the original data set. CHP files generated by the Expression Console Software were used in the Transcriptome Analysis Console (TAC) Software to perform statistical analysis and obtain a list of differentially expressed genes. To run the TAC software, a library folder containing the annotation files was required (HTA-2_0.na36.hg19.probeset.csv) and installed. CHP files were imported and separated into different condition groups for analysis using the Gene Level Differential Expression Analysis tool. A master table was generated containing signal expression levels of all 67528 transcript clusters (genes) covered in the arrays, 44699 protein coding and 22829 non-protein coding. Signal intensity for each transcript cluster was presented as a Bi-weight average signal (log2) value and depending on which two conditions were compared the values of ANOVA p-value, FDR p-value (based on Benjamini-Hochberg Step-Up FDR-controlling Procedure) and gene fold change (linear) were adjusted accordingly.

### Transcriptome analysis of sequencing data from lung biopsy samples

Previously reported sequencing data obtained from the biopsies of 19 control and 20 IPF lungs (GSE92592)^32^ were downloaded from Sequence Read Archive (SRA) (https://www.ncbi.nlm.nih.gov/sra) using the following command in SRA tools: fastq-dump −I–split-files <file_name>. The paired end reads were aligned to the human reference genome (hg38) using Hisat2 (version 2.0.4)^58,59^ using the following command line options: hisat2 −q–dta–rna-strandness FR −x < reference-genone.gtf > −1 < forward_strand.fa > −2 < reverse-strand file.fa > −S < output.sam > . Output SAM files were then sorted and converted to BAM files (samtools sort −@ 8 −o output.bam output.sam) and indexed (samtools index −b output.bam) in Samtools^60^. The profile of gene expression (using the Gencode v27 database) in the BAM files for each samples were determined using Stringtie^61^: stringtie < sample.BAM > −G < GenCodev26.gtf > −1o < samples.gtf > −e −A < sample.txt > . Following feature counting: featureCounts -a < reference-genome.gtf > −g gene_name -o counts.txt Control_*.bam IPF_*.bam the differential gene expression was assessed using DeSeq2 and the following R script: curl −s -O http://data.biostarhandbook.com/rnaseq/code/deseq 2.r cat simple_counts.txt | Rscript deseq2.r 19 × 20 > results_deseq2.txt.

### Chromatin immunoprecipitation, sequencing and analysis of H3K4me1

Chromatin immunoprecipitation (ChIP) using a H3K4me1 antibody (Diagenode, C15410037) and the iDeal ChIP-seq kit for Histones kit (Diagenode, C01010051) was performed on the 5 control and 5 IPF fibroblast samples. In addition, we performed ChIP on a single control and IPF using an IgG antibody to provide a background control. Paired-end 75 bp sequencing data was obtained using the Illumina HiSeq. 4000 at the Oxford Genomics Centre at the Wellcome Centre for Human Genetics (funded by Wellcome Trust grant reference 203141/Z/16/Z). FASTQ sequencing data from control (n = 5) and IPF (n = 5) fibroblasts was aligned to hg38 using Bowtie 2^62^: bowtie2 −q–very-fast < reference_genome.gtf > −U < file_name.fastq > −S < output_file.sam > . Output SAM files were then sorted and converted to BAM files (samtools sort −@ 8 −o output.bam output.sam), indexed (samtools index −b output.bam) in Samtools^60^ and then converted to BigWig format using BamCoverage (which is part of the deepTools suite^63^) using the following command line: bamCoverage −b < input_bam.bam > –normalizeUsingRPKM–binSize 30–smoothLength 300 -p 10–extendReads 200 −o < output_file.bw > . Significant ChIPseq peaks (q =< 0.1) in each sample were called with MACS2^64^ using the broadpeak options: macs2 callpeak –t < sample > −c < backgrounf_igG > −broad − < output_files > −g hs. The differential expression of H3K4me1 peaks was determined by inputing the individual Bam files (Bowtie2) and BED files (Broadpeaks output - MACS2) for control and IPF samples into Diffbind (version 2.2.1) on Galaxy (at www.usegalaxy.org)^65^. Control 1 was omitted as an outlier following PCA analysis.

### Principle Component Analysis and Hierarchical Clustering

The abundance of Gencode v27 defined genes in individual samples was defined as the fragments per kilobase exon per million reads mapped (FPKM) and determined using Stringtie (RNA). PCA and hierarchical clustering on Gencode v27 genes demonstrating an expression > 1 FPKM was performed using Genesis (v1.7.7)^66^. Data was log2 transformed following the addition of 1 FPKM. The threshold for reporting gene expression at FPKM > 1 is based upon the ability to validate sequencing data using qRT-PCR.

### Statisical Analysis

All statistical analysis and graphs were generated using GraphPad Prism 7 software.

## Supplementary information

Dataset 1 Table showing ChIPseq H3K4me1 binding in control and IPF fibroblasts (MOESM1)

Dataset 2 Images of original Western blot gels showing expression of H3K4me, H3K4me 3 and H3K27ac in control and IPF fibroblasts (MOESM2)

Dataset 3 Overall microarray data showing gene expression in non-stimulated and TGFß-stimulated control and IPF fibroblasts (MOESM3)

Dataset 4 Differential gene expression in TGFß-stimulated control fibroblasts (MOESM4)

Dataset 5 Differential gene expression in TGFß-stimulated IPF fibroblasts (MOESM5)

Dataset 6 Differential gene expression between TGFß-stimulated control and IPF fibroblasts (MOESM6)

Dataset 7 Differential gene expression between non-stimulated control and IPF fibroblasts (MOESM7)

Dataset 8 Differential gene expression between control and IPF biopsies (MOESM8)

## Data Availability

The microarray and ChIPSeq sequencing data is available from the gene expression omnibus under GSE129164 and GSE129085. The RNA data for control and IPF biopsies is available at GSE92592.
