# Supplementary material for: Long intergenic non-coding RNAs regulate human lung fibroblast function: Implications for idiopathic pulmonary fibrosis
Source: Sci Rep. 2019 Apr 15;9:6020. doi: 10.1038/s41598-019-42292-w (PMC6465406; doi:10.1038/s41598-019-42292-w)

Long intergenic non-coding RNAs regulate human lung fibroblast function: Implications for idiopathic pulmonary fibrosis

Marina R. Hadjicharalambous, Benoit T. Roux, Eszter Csomor, Carol A. Feghali-Bostwick, Lynne A. Murray, Deborah L. Clarke and Mark A. Lindsay

Supplemental Figure 1 – Original gel images showing labelling (in red) of H3K4me1, H3K4me3 and H3K27ac in control (n=5) and IPF (n=5) lung fibroblasts. Histone marks were quantified using beta-actin as an internal positive control (green)

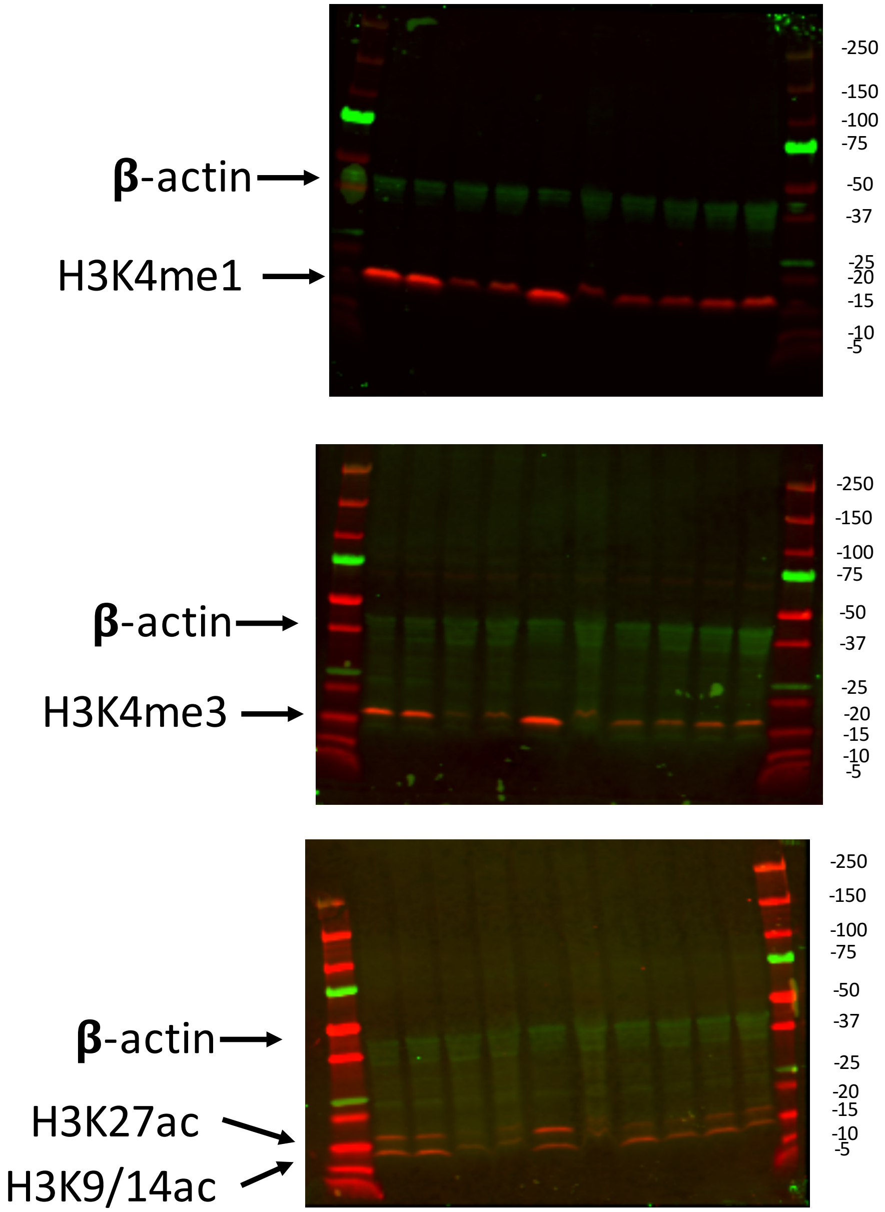

Supplement: Supplementary file 2 — Dataset 2 Images of original Western blot gels showing expression of H3K4me, H3K4me 3 and H3K27ac in control and IPF fibroblasts (MOESM2) [file 41598_2019_42292_MOESM2_ESM.pdf]
